# Surgical treatment for left atrial rupture due to myxomatous mitral valve disease in three dogs: A case report

**DOI:** 10.1002/vms3.915

**Published:** 2022-08-28

**Authors:** Tomohiko Yoshida, Katsuhiro Matsuura, Cheng Chieh‐Jen, Yuki Aboshi, Shusaku Yamada, Hideki Yotsuida, Mizuki Hasegawa, Hanan A. Hendawy, Hussein M. El‐Husseiny, Yusuke Takahashi, Yuki Oonuma, Youta Yaginuma, Shou Fukuzumi, Ryou Tanaka

**Affiliations:** ^1^ VCA Japan Shiraishi Animal Hospital Saitama Japan; ^2^ Department of Veterinary Surgery Tokyo University of Agriculture and Technology Tokyo Japan; ^3^ Department of Clinical Engineering National Cerebral and Cardiovascular Center Osaka Japan; ^4^ Faculty of Veterinary Medicine Department of Surgery Anesthesiology, and Radiology Benha University, Moshtohor, Toukh, Elqaliobiya Egypt

**Keywords:** cardiac surgery, cardiopulmonary bypass, left atrial rupture, mitral valve plasty

## Abstract

**Introduction:**

Myxomatous mitral valve degeneration (MMVD) is an acquired heart disease which sometimes result in pulmonary oedema and left atrial rupture. In previous reports, left atrial rupture has been non‐surgically controlled and its prognosis investigated. There is, however, no report concerning surgically treated left atrial rupture with mitral valvuloplasty and follow‐up results.

**Objectives:**

This report aimed to develop a surgical strategy for a case of left atrial rupture caused by MMVD.

**Materials and methods:**

Three dogs were presented at a private hospital for surgical treatment of MMVD. All three dogs had a previous history of left atrial rupture due to MMVD. The left atrium rapture was diagnosed from indicating that characteristics of the drained pericardial effusion consistent with blood. Mitral valvuloplasty was performed in all dogs using an extracorporeal circulation machine, and the surgical procedure was modified according to each case. In cases with severe adhesion between the pericardial and left atrial appendage, suturing of the left atrial appendage was performed strategically. Additionally, in cases with severe hypotension caused by left atrial rupture, cardiopulmonary bypass was started as soon as possible during the surgical procedure.

**Discussion and Conclusion:**

Since the haemodynamics of all dogs had improved, and the owner reported no cardiac‐related clinical signs, all drugs were withdrawn 3 months after surgery. Since left atrial rupture due to MMVD can cause hypotension, cardiopulmonary bypass should be started as soon as possible during the surgical procedure to maintain the blood pressure and suturing of the left atrial appendage should be performed strategically.

## Graphical abstract

Mitral valvuloplasty was performed for three dogs which diagnosed left atrial rupture due to myxomatous mitral valve degeneration (MMVD). Since left atrial rupture due to MMVD can cause hypotension, cardiopulmonary bypass should be started as soon as possible during the surgical procedure to maintain the blood pressure and suturing of the left atrial appendage should be performed strategically.

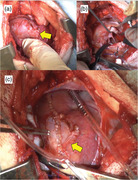

## 1 INTRODUCTION

Myxomatous mitral valve degeneration (MMVD) is an acquired heart disease that commonly causes left‐sided congestive heart failure (CHF) in dogs (Buchanan, 1977; Serres et al., 2007). The prevalence of MMVD increases markedly with age in small breed dogs and if the regurgitation continues to worsen, increased left atrial pressure will result in pulmonary oedema and sometimes left ‐atrial rupture (Buchanan, 1972; Fox, 2012; Nakamura et al., 2014; Sadanaga et al., 1990). Although left atrial rupture has been reported as a rare consequence of MMVD in dogs, left atrial rupture can cause rapid bleeding and cardiac tamponade, and in some cases sudden death (Buchanan, 1972; Fox, 2012; Nakamura et al., 2014). Sometimes, as a result of left atrial rupture, left atrial pressure may decrease and bleeding may stop. However, the underlying mitral valve disease is progressive and most patients will die of recurrent CHF or complications during medical treatment (Serres et al., 2007). In previous reports, left atrial rupture has been nonsurgically controlled and its prognosis investigated (Reineke et al., 2008). Medical treatment can improve dog's condition temporarily. However, due to medical treatment for left atrial rupture being palliative unless the MMVD is radically cured, MMVD remains progressive with a poor prognosis. There is, however, no report concerning surgically treated left atrial rupture and follow‐up results. Here we present three cases of left atrial rupture caused by MMVD, in which Mitral valvuloplasty (MVP) was performed. Depending on the site of rupture and pericardial adhesion, the surgical procedure was adjusted to cope with each specific situation. All three cases achieved long‐term survival, with complete cessation of cardiac medication.

## 2 CASE DESCRIPTION

All three dogs were presented to private hospital for surgical treatment of MMVD. Table 1 summarises the following clinical characteristics of each dog: age, sex, breed, body weight, cardiac murmur, ACVIM stage classification, VHS and VLAS.

**TABLE 1:** Perioperative clinical characteristics of the three dogs

	Dog 1	Dog 2	Dog 3
Age, years	9	10	10
Sex	sF	sF	sF
Breed	Chihuahua	Chihuahua	Chihuahua
BCS	III/V	III/V	III/V
Body weight, kg	4.3	3.14	4.7
Cardiac murmur (grade)	V/VI	IV/VI	V/VI
ACVIM stage	D	C	D
VHS	11.5 v	10.9 v	12 v
VLAS	3.3 v	3.4 v	3.2 v
CTR, %	70 %	75 %	76 %
From the first visit, the day when the left atrium ruptured (days)	1	21	Unclear
Concurrent disease after MVP	None	None	None
Hospitalisation (days)	6	7	7

Abbreviations: CTR, cardio‐thoracic ratio; MVP, mitral valvuloplasty; VHS, vertebral heart size; VLAS, vertebral left atrial size; sF, spayed female.

Case 1 was presented for MVP having experienced past episodes of pulmonary oedema (Day 1). Echocardiography was first attempted as a part of a preoperative evaluation. However, the dog collapsed during the examination and required stabilising with oxygen therapy. Echocardiography several minutes after the episode revealed the presence of pericardial effusion. Together with signs of elongated CRT >2 s and right atrial collapse, a diagnosis of cardiac tamponade was made and pericardial drainage was performed. The drained pericardial effusion exhibited characteristics consistent with blood (Ht 30%, HGB 12.3 g/dl). Upon pericardial drainage and oxygen therapy, the cardiac tamponade was relieved and blood pressure and CRT improved. Echocardiographic variables after relieving the cardiac tamponade are shown in Table 2. The dog did not have anaemia (Ht 44.4 %, HGB 15.2 g/dl), though serum biochemical parameters revealed increased blood urea nitrogen (38.2 mg/dl, reference range: 9.2–29.9 mg/dl). The dog was prescribed pimobendan 0.3 mg/kg, BID (Pimobeheart, Kyoritsu Seiyaku Corporation, Tokyo, Japan), furosemide 1.5 mg/kg, BID (Furosemide, Nipro Corporation, Oosaka, Japan), and temocapril 0.5 mg/head, SID (Pimobeheart, Kyoritsu Seiyaku Corporation, Tokyo, Japan). The case remained stable after cardiac tamponade drainage and MVP was performed on Day 11.

**TABLE 2:** Conventional echocardiography of the three dogs, carried out preoperatively, and at 1 month and 3 months postoperatively

				Postoperative					
	Preoperative			1 month			6 months		
Parameter	Case 1	Case 2	Case 3	Case 1	Case 2	Case 3	Case 1	Case 2	Case 3
BW, kg	2.05	2.98	4.66	2.2	2.9	4.75	2.3	3.1	4.8
HR, bpm	156	189	166	128	90	108	121	95	110
LVIDd, mm	30.2	29.4	32.1	24.4	18.9	28.3	22.1	17.9	24.8
LVIDs, mm	14.5	11.7	17.6	14.8	13.1	20.9	13.3	11.8	18
LVIDDN	2.44	2.13	2.04	1.93	1.39	1.78	1.72	1.28	1.56
LA/Ao	2.22	2.49	1.98	1.81	1.37	1.7	1.65	1.5	1.38
FS, %	52	60.4	45	39	30.5	26	40	32.9	27.4
LVOT, cm/s	113	118	111	98	126	100	95	115	108
E wave, cm/s	128	122	115	88	77.4	95	85	81	88
A velocity, cm/s	69.8	118	85	110	122	100	105	110	95
E/A	1.83	1.03	1.35	0.8	0.59	0.95	0.8	0.73	0.92
E’ sep, cm/s	5.5	6.3	6.8	4.3	5.3	5.7	4.1	5.0	4.5
E’ lat, cm/s	6.1	7.2	7.5	5.1	5.6	6.5	4.5	5.2	4.7
E/E’ sep	23.2	19.4	16.9	20.4	14.6	16.6	20.7	16.2	19.5
E/E’ lat	20.9	16.9	15.3	17.2	13.8	14.6	18.9	15.6	18.7
BUN, mg/dl	38	53	36	28	33	22	22	26	26
CRE, mg/dl	1.7	1.9	1.6	1.4	1.5	1.4	1.4	1.4	1.3

Abbreviations: A velocity, peak velocity of late diastolic transmitral flow; BW, body weight; E/A, the ratio of peak velocity of early diastolic transmitral flow to peak velocity of late diastolic transmitral flow; E velocity, peak velocity of early diastolic transmitral flow; E', early diastolic wave signal as measured by Tissue Doppler imaging; FS, fractional shortening; HR, heart rate; LA/Ao, the ratio of the left atrial dimension to the aortic annulus dimension; lat, mitral annulus at the left ventricular lateral wall; LVIDd, left ventricular end‐diastolic diameter; LVIDDN, left ventricular end‐diastolic internal diameter normalized to body weight; sep, mitral annulus at the septal wall.

*Note*: LVIDD was standardised by dividing LVIDD to the body weight (BW) raised to the 0.294 power (Cornell et al., 2004). LVIDDN was calculated using an allometric approach.

Case 2 was scheduled for elective MVP surgery. Prior to the day of surgery, no pericardial effusion was recognised on echocardiography (Day 1). Echocardiographic variables are shown in Table 2. Pimobendan 0.5 mg/kg, BID (Pimobeheart, Kyoritsu Seiyaku Corporation, Tokyo, Japan), torasemide 0.25 mg/kg, BID (Furosemide, Nipro Corporation, Osaka, Japan), and hydrochlorothiazide 0.5 mg/kg, BID (ACEWORKER; Kyoritsu Seiyaku Corporation, Tokyo, Japan) were prescribed before operation. On the scheduled day of the MVP (Day 21), the dog collapsed prior to surgery, during intravenous cannulation on the forelimbs. During oxygen supplementation, pericardial effusion was recognised on echocardiography. The left atrium rapture due to MMVD was diagnosed from exhibiting that characteristics of the drained pericardial effusion consistent with blood (Ht 42%, HGB 13.3 g/dl). An emergency operation to identify the haemorrhage site and control the active bleeding was performed. Concomitantly, MVP was performed for the definitive treatment of MMVD.

Case 3 was presented for MVP due to repeated episodes of pulmonary oedema. No pericardial effusion was recognised on echocardiography (Day 1). Echocardiographic variables are shown in Table 2. Pimobendan 0.5 mg/kg, BID (Pimobeheart, Kyoritsu seiyaku Corporation, Tokyo, Japan), torasemide 0.25 mg/kg, BID (Luprac; Mitsubishi Tanabe Pharma Corporation, Osaka, Japan) and hydrochlorothiazide 0.5 mg/kg, BID (Hydrochlorothiazide; Towa Pharmaceutical Co., Ltd., Osaka, Japan) were prescribed. The surgery was scheduled for Day 8. No pericardial effusion was yet recognised on the echocardiography performed on the day of surgery. After thoracotomy, however, adhesion between the pericardium and the left atrial appendage was noticed, and thus previous left atrial rupture episodes were suspected.

The surgical MVP procedures for each dog are detailed below.

### 2.1 Surgical procedure

For Case 1, the surgical steps were followed in the usual order (procedures 1→7) (Yoshida et al., 2022; Yoshida et al., 2021)(reference to below for each procedure description). For Case 2, since the surgery was performed as an emergency operation on the day when the left atrial rupture occurred, procedure 4 had precedence over others. In addition, to maintain blood pressure, a connection to an extracorporeal circulation machine was made as soon as possible (procedures 1→4→2→3→5→6→7). In Case 3, where adhesion between the left atrial appendage and pericardium was recognised, steps involving pericardiectomy, incision and suturing of the left atrial appendage required different techniques from Case 1 and 2. Considering the possibility of haemorrhage during detachment of the adhesion site, connection to the extracorporeal circulation machine was given precedence, as in Case 2(procedures 1→4→2→3→5→6→7). The specific descriptions of each procedure are detailed below.

#### 2.1.1 Procedure 1: Anaesthesia

As premedication, atropine sulphate 0.05 mg/kg SC (Atropine sulphate; Nipro Es Pharma CO., LTD. Osaka, Japan), fentanyl 5 μg/kg, IV (Fentanyl Citrate; Daiichi Sankyo Company, Limited, Tokyo, Japan) and midazolam hydrochloride 0.2 mg/kg, SC (Dormicum; Astellas Pharma Inc, Tokyo, Japan) were administered. Induction of anaesthesia was conducted using 1% propofol 6 mg/kg IV (Propofol Mylan; Mylan Seiyaku, Tokyo, Japan) and maintained with 0.5–2.0 vol% of isoflurane (Isoflurane for Animal Use; Intervet, Osaka, Japan) and fentanyl (Fentanyl Citrate; Daiichi Sankyo Company, Limited, Tokyo, Japan) (10 μg/kg/min). In Case 2, due to blood pressure compromise, premedication with fentanyl and midazolam was suspended.

#### 2.1.2 Procedure 2: Haemodynamic monitoring

The right femoral artery and vein were cannulated to measure arterial and central venous blood pressure.

#### 2.1.3 Procedure 3: Thoracotomy and approach to the left atrium

**FIGURE 1:**
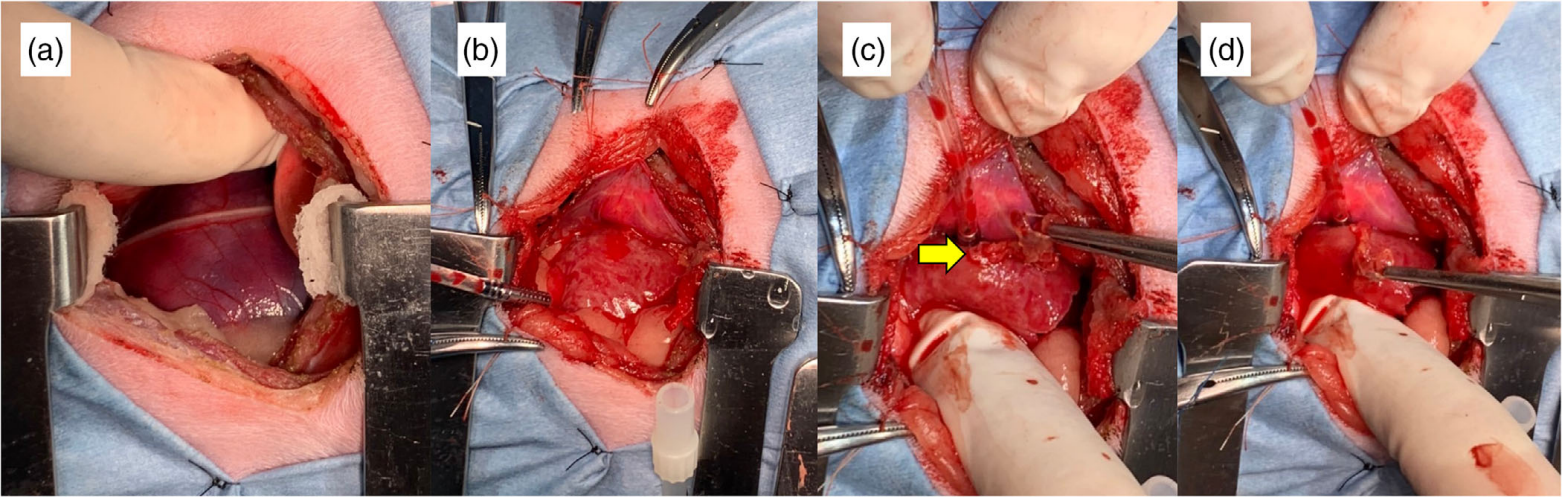
Intraoperative images of Case 2 with haemorrhage due to left atrium rupture. (a) Intrapericardial haemorrhage was recognised. (b) The images of left atrial appendage after incising pericardial. (c, d) The rupture site was identified by pulling the left atrial appendage. Yellow arrow indicates the site of haemorrhage. The clot was grasped with forceps

**FIGURE 2:**
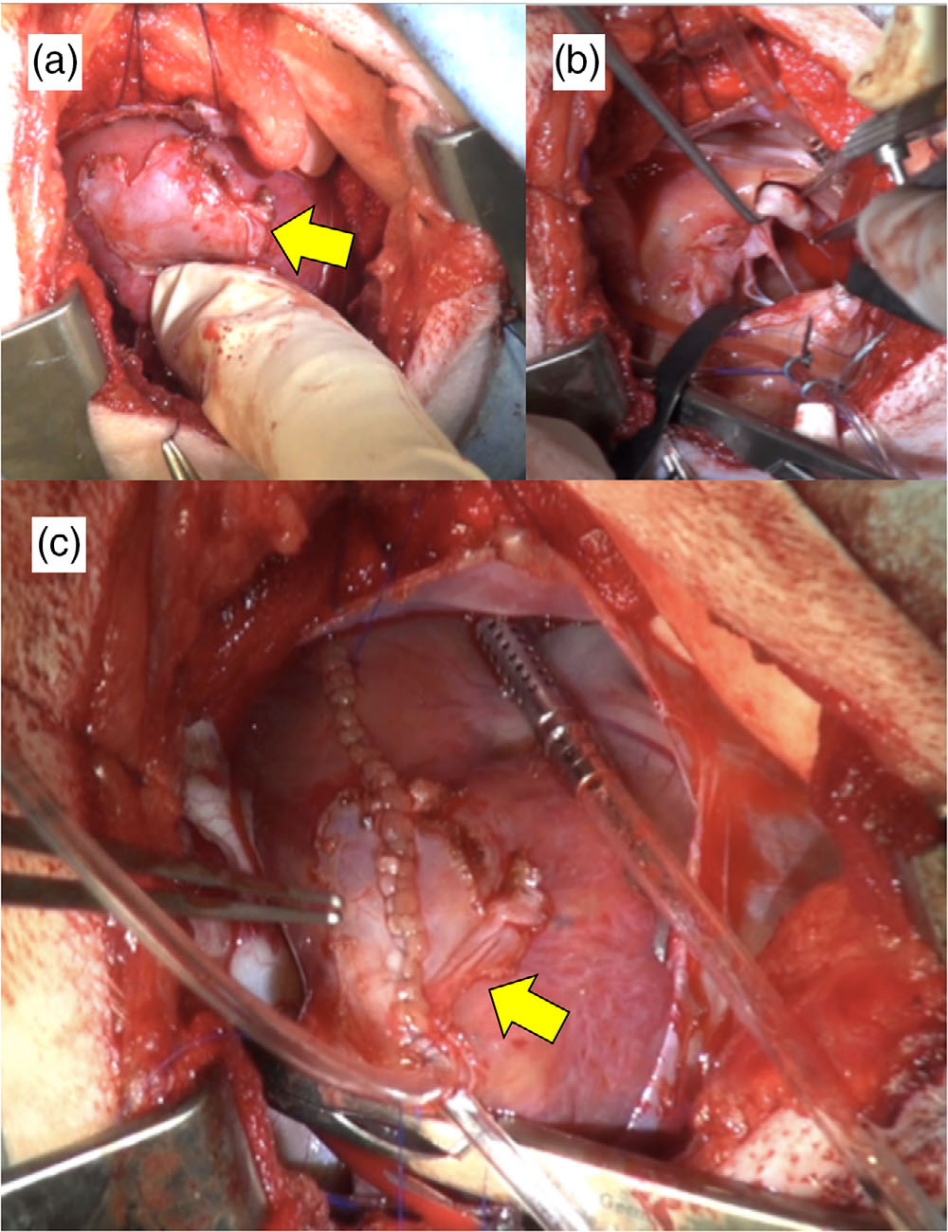
Intraoperative images of Case 3. (a) Adhesion between the pericardium and the left atrial appendage. (b) The incision was made on the pericardium‐adhered area of the left atrial appendage, with MVP then performed as usual. (c) Closure of the incised adhesion site of the left atrial appendage. Yellow arrow indicates adhesion between the pericardium and the left atrial appendage

After left intercostal thoracotomy, a pericardial incision was conducted to approach the left atrium appendage. In Cases 1 and 2, pericardial effusion was visually identified by thoracotomy. In addition, blood mixed with clots was collected after the pericardiectomy. While the ruptured site of the left atrium was blocked with clotted blood in Case 1, in Case 2, there was slight yet continuous haemorrhage from the rupture (Figure 1a–d). No adhesion between the left atrial appendage and pericardium was recognised in either Case 1 or 2. No adhesion between the left atrial appendage and pericardium was recognised in either Case 1 or 2. In Case 3, the site of the rupture in left atrium was covered with the adhered pericardium (Figure 2a). Due to the severe adhesion between the left atrial appendage and the pericardium, detachment of the adhesion site was not performed.

#### 2.1.4 Procedure 4: Preparing the cardiopulmonary bypass

After heparin sodium administration IV 400 IU/kg IV (Heparin sodium injection 10,000 units/10 ml, Nipro corporation, Tokyo, Japan), the left carotid artery and jugular vein were cannulated to connect to the cardiopulmonary bypass circuit.

#### 2.1.5 Procedure 5: Mitral valvuloplasty

Cardiac arrest was achieved by aortic clamping followed by the administration of cardioplegia (Miotecter, KYOWA CritiCare Co., Ltd., Tokyo, Japan) from the aortic root cannula, and the cardiopulmonary bypass was started. After the incision on the left atrium appendage, mitral valve reconstruction and suture annuloplasty were performed using artificial chordae tendineae (ePTFE, GORE‐TEX®, W. L. Gore & Associates, Inc., Newark, USA; Figure 2b). In Case 2, since the ruptured site coincided with the scheduled incision line of the left atrium, the incision was made directly above the ruptured site. In Case 3, since the left atrial appendage and the pericardium had adhered, both the left atrial appendage and the pericardium were cut up together with a single incision.

#### 2.1.6 Procedure 6: Suturing the left atrial appendage

After mitral valve reconstruction and suture annuloplasty, suturing of the left atrial appendage was performed. In Cases 1 and 2, closure of the left atrial appendage was performed by continuous suture as usual. In Case 3, the left atrial appendage was closed together with the adhered pericardium by continuous suture (Figure 2c).

#### 2.1.7 Procedure 7

After terminal warm coronary reperfusion and return of the sinus rhythm, the aortic clamping was released. The patient was weaned from cardiopulmonary bypass before modified ultrafiltration. After a protamine infusion to antagonise the effect of heparinisation, the chest was closed in the usual manner.

**FIGURE 3:**
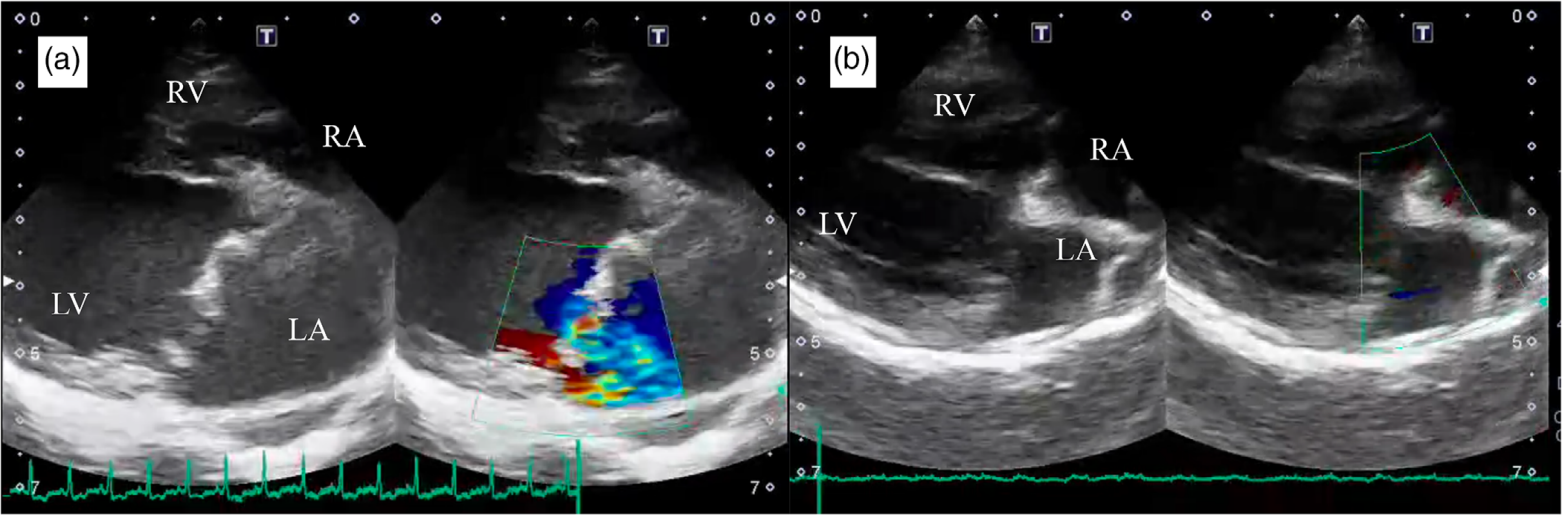
Representative echocardiogram images before and after mitral valvuloplasty in Case 1. (a) Two‐dimensional echocardiography at the long‐axis LV inflow view before mitral valvuloplasty. The enlarged atrioventricular lumen size and severe mitral regurgitation were recognised. (b) Atrioventricular lumen size and amount of mitral regurgitation were reduced after mitral valvuloplasty. LA, left atrium; LV, left ventricle; RA, right atrium; RV, right ventricle

After surgery, all three cases underwent an uneventful recovery under 24‐h intensive care, involving the monitoring of vital signs, haemodynamics and urine output. Patients were discharged on Days 7–10 after operation, with pimobendan 0.35 mg/kg, BID (Vetmedin, Boehringer Ingelheim, Ingelheim, Germany), rivaroxaban 1.0 mg/kg, SID (Xarelto, Bayer Yakuhin, Ltd., Osaka, Japan) and enrofloxacin 5 mg/kg, SID (Baytril, Bayer Yakuhin, Ltd., Osaka, Japan) for postoperative antibiotic prophylaxis. All dogs had follow‐up evaluations by echocardiography and blood examination on Days 7 and 14 and Months 1, 2, 3 and 6 after discharge (Table 2). Figure 3 is echocardiogram images in Case 1 before and after MVP. Since the haemodynamics of all dogs were improved and the owner reported no cardiac‐related clinical signs, all drugs were withdrawn 3 months after surgery. The condition of all dogs remained good for at least 6 months after surgery (Table 2).

## 3 DISCUSSION

As a complication of MMVD, canine left atrial rupture has been reported to have a poor prognosis. Reineke et al. reported that among the 14 dogs diagnosed with left atrial rupture secondary to MMVD, one dog died on arrival, one presented with cardiac arrest immediately after the onset of clinical signs, five were euthanised during the first hospitalisation and three were euthanised within 35 days of diagnosis (Nakamura et al., 2014). On the other hand, the survival time of dogs that survived the acute phase of left atrial rupture has been extended, to some degree, through medical management (Reineke et al., 2008). The possibility of recurrence remains, however, if the underlying cause is not addressed. Here, we have performed surgical treatment in a case where the left atrial rupture occurred on the day of operation, and two cases where the left atrial rupture was medically controlled before surgery. The surgical results of all three cases turned out to be uneventful, with no complications, and withdrawal of all medications 3 months after surgery was achieved. Mitral valvuloplasty solved the underlying cause of left atrial rupture by reducing the load on the left atrium, which results in improving the condition of the three patients. However, there are several points to note when performing surgery on MMVD cases complicated with left atrial rupture. The first concerns the risk of anaesthesia. In cases presented with left atrial rupture, the underlying MMVD is usually severe, meaning an abrupt change in the patient's condition during induction of anaesthesia may occur. In Case 2, where the surgery was performed just after left atrial rupture, the increased risk from anaesthesia led to the decision to cease fentanyl administration as a premedication. We recommend the routine, careful monitoring of blood pressure during the induction of anaesthesia when performing MVP in cases with left atrial rupture. Second, there is an increased risk of haemorrhage during surgery. Emergency surgery in cases with left atrial rupture demands swift connection to the extracorporeal circulation machine in order to maintain the blood pressure. In Case 2, the acute nature of the left atrial rupture that occurred immediately before surgery required even earlier connection to the extracorporeal circulation machine. In Case 3, concerns regarding the risk of haemorrhage and subsequent decrease in blood pressure during detachment of adhered pericardium led to the decision to prioritise the extracorporeal circulation connection. However, early extracorporeal circulation connection necessitated even earlier heparin administration, that is, immediately after the procedure began, which also increased the risk of haemorrhage. In the standard operation, heparin is usually given after thoracotomy is performed (Fox, 2012; Suzuki et al., 2020; Uechi, 2012). Third, surgical difficulty may increase if adhesion between the left atrial appendage and pericardium is present due to left atrial rupture. What makes Case 3 unique is that adhesion between the left atrial appendage and pericardium necessitated different approaches to incise and suture the left atrial appendage than those of Cases 1 and 2. The aggressive removal of the adhered pericardium might have resulted in haemorrhaging from the left atrial appendage. Therefore, instead of detachment, trimming around the adhered pericardium was performed. Also, the left atrial appendage incision and closure were conducted with the adhered pericardium at once. If the rupture and surgical sites were not consistent, purse‐string suturing might have been necessary. In this report, we surgically corrected left atrial rupture cases caused by MMVD. It is hard to judge whether a surgical correction should be immediately performed at the time the atrial rupture is found. Provided the client is obtained, and immediate surgery is available, surgery is considered an effective treatment option as well. Nevertheless, in general, as the availability of surgery is limited, medical management before surgical correction is also essential.

In conclusion, this study reports the therapeutic strategy for dogs with left atrial rupture due to severe MMVD. To fundamentally treat left atrial rupture, mitral valve reconstruction and suture annuloplasty were performed under cardiopulmonary bypass. Our findings suggest that in cases with severe adhesion between the pericardial and left atrial appendage, suturing of the left atrial appendage should be performed strategically. In addition, cardiopulmonary bypass should be started as soon as possible during the procedure.

## AUTHOR CONTRIBUTIONS

T.Y. and K.M. designed the report and drafted the manuscript. Y.A., S.Y., H.Y. and M.H. acquired and analysed data. C.C, Y.O., Y.T. and H.H. critically reviewed the manuscript. Y.Y., S.F. and M.W. did data management, data interpretation and manuscript preparation was carried out by R.T. and H.E.

## CONFLICT OF INTEREST

The authors declare there is no conflict of interest.

## FUNDING

The authors received no specific grant from funding agencies in the public, commercial or not‐for‐profit sectors.

### ETHICS STATEMENT

Written informed consent authorizing the participation of the case was obtained from the dog's owner.

### PEER REVIEW

The peer review history for this article is available at https://publons.com/publon/10.1002/vms3.915.

## Data Availability

The raw data supporting the conclusions of this article will be made available by the authors, without undue reservation.
